# Genome-wide association study of footrot in Texel sheep

**DOI:** 10.1186/s12711-015-0119-3

**Published:** 2015-04-30

**Authors:** Sebastian Mucha, Lutz Bunger, Joanne Conington

**Affiliations:** Animal and Veterinary Sciences, Scotland’s Rural College, Easter Bush, Midlothian, EH25 9RG, Scotland UK

## Abstract

**Background:**

This is the first study based on a genome-wide association approach that investigates the links between ovine footrot scores and molecular polymorphisms in Texel sheep using the ovine 50 K SNP array (42 883 SNPs (single nucleotide polymorphisms) after quality control). Our aim was to identify molecular predictors of footrot resistance.

**Methods:**

This study used data from animals selected from a footrot-phenotyped Texel sheep population of 2229 sheep with an average of 1.60 scoring records per animal. From these, a subset of 336 animals with extreme trait values for footrot was selected for genotyping based on their phenotypic records. De-regressed estimated breeding values (EBV) for footrot were used as pseudo-phenotypes in the genome-wide association analysis.

**Results:**

Seven SNPs were significant on a chromosome-wise level but the association analysis did not reveal any genome-wise significant SNPs associated with footrot. Based on the current state of knowledge of the ovine genome, it is difficult to clearly link the function of the genes that contain these significant SNPs with a potential role in resistance/susceptibility to footrot. Linkage disequilibrium (LD) was analysed as one of the factors that influence the power of detecting QTL (quantitative trait loci). A mean LD of 0.20 (r^2^ at a distance of 50 kb between two SNPs) in the population analysed was estimated. LD declined from 0.15 to 0.07 and to 0.04 at distances between two SNPs of 100, 1000 and 2000 kb, respectively.

**Conclusions:**

Based on a relatively small number of genotyped animals, this study is a first step to search for genomic regions that are involved in resistance to footrot using the ovine 50 K SNP array. Seven SNPs were found to be significant on a chromosome-wise level. No major genome-wise significant QTL were identified.

## Background

Footrot is an endemic disease of sheep that causes pain, reduced productivity and economic loss to the sheep industry in the United Kingdom (UK) as in other sheep-producing countries in the world. It is a highly contagious disease caused by *Dichelobacter nodosus*, which is the main component of a mixed bacterial infection and is the major cause of lameness in sheep. The annual economic impacts of footrot are considerable; direct costs include the labour time needed to manage the disease as well as prophylactic and veterinary medicine treatment costs. Indirect costs of reduced fertility, milk supply and productivity are less obvious but lame sheep are less productive, have less milk and rear fewer lambs compared to unaffected ewes. The costs of footrot have been estimated to be $18.4 M [1], £24 M [2] and Rs15.82 [3], for Australia, the UK, and central Kashmir India, respectively.

Reports indicate that at least three million sheep are lame in the UK at any one time and that six to nine million sheep become lame in the UK over the course of a year [4]. Prevalence levels of footrot and other hoof lesions in the UK and Ireland, as determined by hoof inspection have been reported by [5] and [6]. Depending on breed type and management system, the average prevalence levels ranged from 13 to 23%, although some individual farms within that study had over 59% of animals with at least one hoof with clinical signs of footrot while one other had less than 0.5%. These figures are in line with prevalence levels reported in New South Wales (Australia) [7] but are higher than the levels of 10% from a self-reporting survey of UK farmers [8], 6% in Swedish sheep [9] and 12% reported in central Kashmir India [3]. The management and breeding opportunities available to control footrot were recently reviewed and covered the aetiology, risk factors and control methods including host resistance [10,11].

The potential for exploiting the host’s genetic resistance to footrot was reported by [12] and reviewed recently by [11]. Evidence for the genetic basis of host resistance to footrot was found both between- and within-breeds [6,13-18]. In general, these studies showed low to moderate heritabilities for footrot and therefore possibilities exist for genetic improvement following screening protocols and conventional quantitative selection strategies. It is important to note that the heritability estimates for affected flocks are higher than for flocks with a low incidence of footrot, which implies that exposure to footrot is important to be able to quantify the genetic potential of an animal to breed more resistant sheep to footrot [19]. Indeed, the footrot scoring protocol described in [18] has now been included in the sheep breeding strategy in Ireland to generate estimated breeding values (EBV) for footrot, using economic values derived by [20]. However, the development and use of a molecular genetic screening for footrot resistance has potentially considerable advantages. First, animals under selection do not need to be exposed to the disease to determine whether they are genetically susceptible or not. Second, the time required and practical difficulty of objectively scoring feet lesions well, and the difficulty of classifying them appropriately, objectively and repeatably can be reduced. Using genotype information offers a practical alternative to laborious phenotypic scoring and disease exposure protocols. Molecular techniques to identify resistant animals would help breeders select for footrot resistance. To this end, studies based on the use of genetic markers that are within resistance genes have been undertaken including the major histocompatibility complex (MHC) located on ovine chromosome 20 [21-24]. In the UK, studies have investigated the use of the 7-point footrot sensitivity test that was developed in New Zealand for class II MHC genes (*DQA2*) in the region between 44 and 55 cM in Texel, Blackface and Welsh Mountain sheep and have shown that the *DQA2* gene is very polymorphic but that the classification of animals into the same footrot sensitivity classes (New Zealand classification) was not informative for these breeds. However, a multiplexed fluorescent microsatellite marker set that covered chromosome 20 at 10 cM intervals was also developed during the project and revealed breed-specific relationships of footrot resistance with genotype. The advent of the genome-wide screening approach using single nucleotide polymorphism (SNP) technology facilitates the search for genes that are involved in resistance to footrot by using SNPs that are significantly associated with the trait. The ovine 50 K SNP chip has been available since 2008 and is used in New Zealand and Australia for key production traits to improve the accuracy of EBV [25,26]. However, linking footrot phenotype to SNPs has not yet been undertaken in the UK. The main aim of this study was to undertake a genome-wide association study (GWAS) to investigate if existing significant SNPs are related to footrot in Texel sheep.

## Methods

### Scoring method and traits

A 5-point scoring method for hoof lesions that follow the progression of footrot was described elsewhere [27], and more recently was validated for use in the UK [18]. This method differentiates clean, unaffected hooves (score 0) from those with mild inter-digital inflammation, (score 1), to inter-digital necrosis (score 2), to some under-running of the sole of the hoof (score 3) and fully under-run to the abaxial wall of the hoof (score 4). Each separate hoof (left and right hind, left and right fore) was screened and hence each animal had a potential maximum total footrot score of 16 per scoring event, with 16 being the worst score (SUM_FR). This latter trait was the phenotype used to derive estimated breeding values (EBV) for the genomic association analyses. Data were collected by two trained technical staff in 2006 and 2007 from 17 commercial farms across the UK on Texel sheep that belong to the national performance recording scheme, *Sheepbreeder* [28]*.* All farms were requested not to treat their sheep for footrot in the 4-week period before the farms were visited.

### Dataset, records and traits

A total of 3573 records were obtained from 2229 animals i.e. 2875 ewes of mixed age and 698 lambs that were born between 1998 and 2006. The sheep were scored between one and three times within an 18 month period averaging 1.60 records per animal. On average, there were between 84 and 127 animals per farm for each scoring event. The average age of the lambs at the time of footrot scoring was 158 days. A total of 11 048 individuals were included in the pedigree file with 2723 sires, 6642 dams and 2165 founders. The maximum pedigree depth was 22 generations with a mean of nine generations. Heritability of footrot in the analysed population was 0.18 (unpublished results).

The first scoring event was carried out during late summer 2006, with scoring dates between 10^th^ July and 12^th^ September, 2006, the second scoring event took place between 1^st^ and 24^th^ May, 2007 when the ewes were in mid-lactation and the third scoring event was between 18^th^ July and 10^th^ September, 2007. The data were generated as part of a collaborative project with the Texel sheep society and could be made available for further analyses subject to the conditions of the original collaboration agreement.

### Genotyping

Blood samples from 336 animals were collected in order to extract DNA. Animals were selected for extreme phenotypes (the highest scores) irrespective of the farm of origin and these were matched with animals with a 0 score from the same farms. The minimum number of genotyped animals per farm was 6 with the majority of farms with more than 10 genotyped animals. Animals with a 0 score were sampled from 15 farms with a minimum of 3 and a maximum of 21 animals per farm. Animals were genotyped commercially with the Illumina ovine SNP50 BeadChip at Ark Genomics (Edinburgh, UK). Animals with extreme phenotypes i.e. with no footrot detected on any of the scoring events (179 animals) or with a score of 8 and above on at least one scoring event (25 animals) were selected for genotyping. This made up the core of the genotyped population consisting of 204 animals which were subsequently combined with 132 animals with intermediate scores (between 1 and 7). The genotyped animals originated from 118 sires and 293 dams. They included 22 full-sib families with two offspring, and 117 half-sib families with an average of 3.2 animals per family.

SNPs were removed from further analyses if they were not in Hardy-Weinberg equilibrium, had a minor allele frequency less than 0.05, were monomorphic, had a call rate less than 0.95 or if the GC score was less than 0.6. Missing genotypes were imputed as homozygous for the major allele.

### Calculation of de-regressed breeding values

Basic statistics describing the data were estimated with R package [29]. After an initial investigation of fixed effects and co-variables, an appropriate statistical model for footrot was determined by stepwise elimination of non-significant interactions and main effects. Breeding values were estimated using all available records (3573) from 2229 animals using the software package MIX99 [30] applying the following model:

$$ \mathbf{y}=\mathbf{X}\mathbf{b}+\mathbf{Z}\mathbf{a}+\mathbf{e}, $$

where **y** is the vector of footrot scores, **b** is the vector of fixed effects consisting of birth year (years, 9 levels), type (adult ewe, lamb), farm (17 levels), and scoring event (3 levels), **a** is the vector of random animal effects, and **e** is the vector of random residuals.

Random effects were assumed to be normally distributed with zero means and the following covariance structure:

$$ Var\left[\begin{array}{c}\hfill \mathbf{a}\hfill \\ {}\hfill \mathbf{e}\hfill \end{array}\right]=\left[\begin{array}{cc}\hfill \mathbf{A}{\sigma}_a^2\hfill & \hfill 0\hfill \\ {}\hfill 0\hfill & \hfill \mathbf{I}{\sigma}_e^2\hfill \end{array}\right], $$

where **A** is the pedigree-based relationship matrix, ${\sigma}_a^2$ is the genetic variance, and ${\sigma}_e^2$ is the residual variance.

The software package MIX99 was also used for de-regression, using a full animal pedigree with effective offspring contributions (EOC) as weighting factors. EOC were calculated as:

$$ EO{C}_i=\frac{re{l}_i\cdot kdau}{1-re{l}_i}, $$

$$ kdau=\frac{4-{h}^2}{h^2}, $$

where *rel*_*i*_ is the reliability of EBV for animal *i* and *h*^2^ is the heritability of footrot.

The de-regressing procedure was performed to eliminate bias in the EBV from animals with different numbers of offspring. De-regressed EBV were used as pseudo-phenotype in the subsequent GWAS analysis in order to maximise the use of available information since 100 of the genotyped animals had progeny with footrot records, and half of the genotyped animals had repeated measurements of footrot (scored two or three times).

### Genome-wide association analysis

GWAS was performed using the Multi-Locus Mixed Model (MLMM) algorithm [31] implemented in SNP & Variation Suite v7.7.8 (Golden Helix Inc., Bozeman, MT). The following model with a random polygenic effect and the genotypes at single SNPs as fixed effects was used:

$$ \mathbf{y}=\mathbf{X}\boldsymbol{\upbeta } +\mathbf{Z}\mathbf{a}+\mathbf{e}, $$

where **y** is the vector of de-regressed EBV for footrot, **β** is a vector of coefficients for the SNP effects, **a** is the vector of random animal effects, **e** is the vector of random residual effects, and **X** and **Z** are incidence matrices relating observations to fixed and random animal effects, respectively. Random effects were assumed to be normally distributed with zero means and the following covariance structure:

$$ Var\left[\begin{array}{c}\hfill \mathbf{a}\hfill \\ {}\hfill \mathbf{e}\hfill \end{array}\right]=\left[\begin{array}{cc}\hfill \mathbf{G}{\sigma}_a^2\hfill & \hfill 0\hfill \\ {}\hfill 0\hfill & \hfill \mathbf{I}{\sigma}_e^2\hfill \end{array}\right], $$

where **G** is the genomic relationship matrix [32] calculated as:

$$ \mathbf{G}=\frac{\mathbf{S}{\mathbf{S}}^{\mathbf{\prime}}}{2{\displaystyle {\sum}_{i=1}^N{p}_i\left(1-{p}_i\right)}}, $$

where **S** is a centred incidence matrix of SNP genotypes, *N* is the number of SNPs, and *p*_*i*_ is allele frequency of marker *i*.

Three different genetic models were used for this study: (1) an additive genetic model where the major homozygous genotype was recoded to 0, the heterozygous genotype to 1, and the minor homozygous genotype to 2; (2) a dominant model, where the minor homozygous and heterozygous genotypes were coded as 1 and the major homozygous genotype was coded as 0; and (3) a recessive model, where the minor homozygous genotype was coded as 1 and the heterozygous and major homozygous genotypes were coded as 0. SNP positions were determined using the map *Ovis aries* OAR 3.1 [33].

### Quantile-quantile plots

Quantile-quantile (Q-Q) plots were used to analyse the extent to which the observed distribution of the test statistic followed the expected (null) distribution. This was done to assess potential systematic bias due to population structure or analytical approach.

### Significance threshold

Bonferroni correction was applied to obtain significance thresholds. A SNP was significant at the genome-wise level when the –log10(p-value) was greater than -log10 (0.05/N), where N is the total number of markers. Chromosome-wise significant SNP had an associated –log10(p-value) above –log10(0.05/n), where n is the number of markers on a given chromosome.

### Proportion of variance explained

Proportion of variance explained by SNP (pve) was calculated as:

$$ pve=\frac{mrs{s}_{h0}-mrs{s}_k}{mrs{s}_{h0}} $$

where mrss_h0_ is the Mahalonobis Root Sum of Squares (mrss) for the null hypothesis and mrss_k_ is the same for marker k.

### Linkage disequilibrium

Linkage disequilibrium (LD) was measured as r^2^, which is the squared correlation of the alleles at two loci [34]:

$$ {r}^2=\frac{{\left[f(AB)-f(A)f(B)\right]}^2}{f(A)f(a)f(B)f(b)}, $$

where *f*(AB), *f*(A), *f*(a), *f*(B), *f*(b) are observed frequencies of haplotype AB and of alleles A, a, B and b, respectively. LD was calculated for all syntenic SNP pairs (SNPs on the same chromosome). SNPs that could not be mapped to any chromosome were excluded from these analyses. Average LD was calculated as an arithmetic mean of r^2^ values for SNP pairs in 1 kb windows from all chromosomes. LD based on the marker data was compared with an approximate expectation of r^2^ [35]:

$$ E\left({r}^2\right)=\frac{1}{\left(4{N}_ec+1\right)}, $$

where N_e_ is the effective population size, c is the recombination distance in Morgans (we assumed 100 Mb = 1 Morgan) between SNPs. Assuming that LD at short distances depends on long-term population history [36,37], the historic effective population size was estimated as:

$$ {N}_t=\frac{1-{r}^2}{4c{r}^2}, $$

where N_t_ is the effective population size t generations ago, with t = 1/(2c) [36]. The Ne from five generations ago (250 individuals) was considered as the most recent with c = 0.1 Morgan.

## Results

### Phenotype statistics and SNP distribution

Descriptive statistics for the footrot score of the animals used in this study are in Table 1. Spearman’s rank correlations were calculated between the three scoring events. The correlation between the first and third scoring event was not significantly different from 0, and the remaining estimates were between 0.09 (first and second scoring event) and 0.15 (second and third scoring event). Trait distribution was strongly skewed with the majority of animals showing no signs of footrot (score of 0). The distribution of de-regressed EBV for the genotyped animals was much closer to the normal distribution ranging from −0.94 to 2.63, with a mean of 0.24. In total, 336 animals were genotyped and three failed quality control. Therefore, 333 animals with de-regressed EBV were used for the GWAS analysis with 42 883 SNPs (after quality control).

**Table 1:** **Number of records (N), number of animals (n), and descriptive statistics for footrot score and de-regressed EBV (dEBV) for footrot**

**Trait**	**N**	**n**	**Mean**	**SD**	**Min**	**Max**	**SEM**
Footrot score^1^	3573	2229	0.68	1.57	0	12	0.03
Footrot score^2^	545	336	1.36	2.41	0	12	0.1
Footrot dEBV	336	336	0.24	0.65	−0.94	2.63	0.04

### Linkage disequilibrium

**Figure 1:**
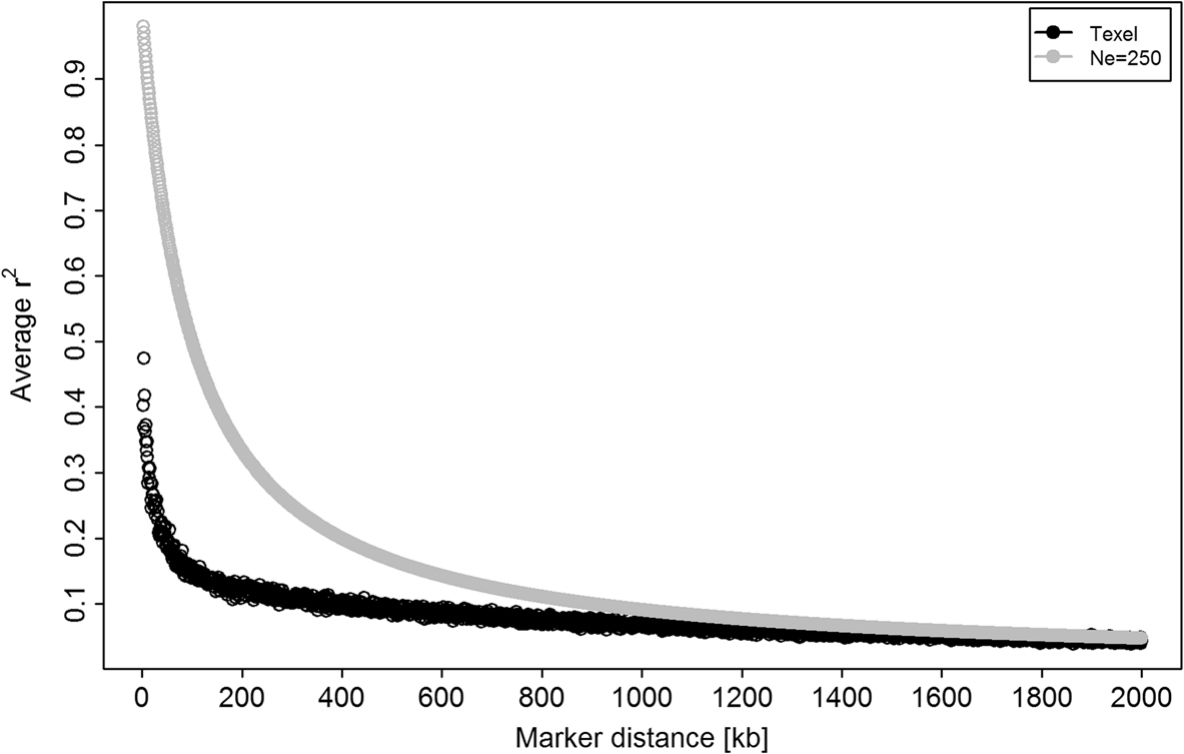
Linkage disequilibrium (r^2^)* calculated for all syntenic SNP pairs. *Calculated as an arithmetic mean of r^2^ values for SNP pairs in 1 kb windows on all chromosomes.

All possible SNP pairs with a distance between SNPs equal to or less than 2000 kb from the 26 ovine autosomes produced 1 351 769 pairwise r^2^. The average r^2^ among syntenic markers (within a 1 kb window) as a function of marker distance is in Figure 1. The largest decline of LD was for distances between two SNPs less than 100 kb. In the studied population, the mean r^2^ at 50 kb (distance between two SNPs) was 0.20. LD declined from 0.15 at 100 kb (distance between two SNPs) to 0.07 at 1000 kb, and 0.04 at 2000 kb. Compared with the expected LD for a population with N_e_ = 250, the r^2^ in the Texel sheep population was lower for small distances between SNPs and similar for large distances (Figure 1).

### Quantile-quantile (Q-Q) plots of genome-wide association results for footrot using additive, dominant, and recessive models

**Figure 2:**
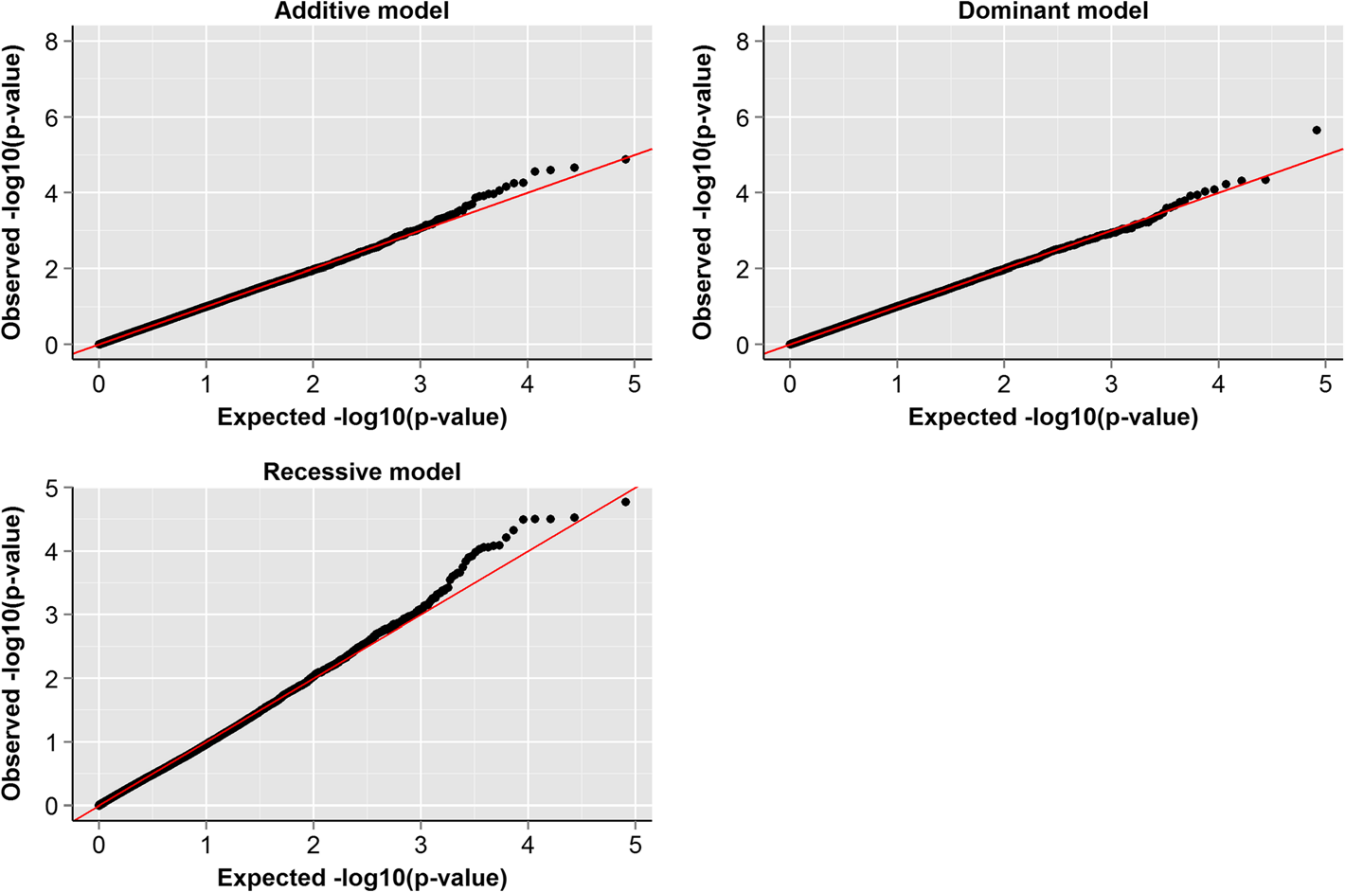
Quantile-quantile (Q-Q) plots of genome-wide association results for footrot using additive, dominant, and recessive models.

Q-Q plots are probability plots and are used as a graphical method to compare two probability distributions by plotting their quantiles against each other. Here, Q-Q plots of expected and observed p-values (−log10 p-values) were constructed but they did not reveal any population stratification that might have affected the current analysis (Figure 2). The Q-Q plots also show that very few SNPs depart from the expected probability, which indicates that there are no markers with a highly significant effect on the analysed trait. Values of the inflation factor lambda for the analysed models were equal to 1.01, 1.00, and 0.98 for the additive, dominance, and recessive models, respectively. Homogeneity of the analysed population was also confirmed with a principal component analysis (results not shown).

### Association analysis

**Figure 3:**
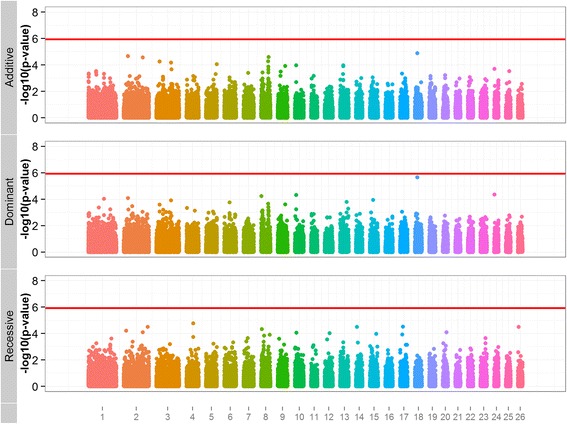
Genome-wide plot of -log10(p-values) for association with footrot score.

Profiles of the p-values (presented as –log10 (p-value)) for all SNPs from chromosomes 1 to 26 are in Figure 3. No markers were significant on the genome-wise level (p < 0.05) after applying the strict Bonferroni correction. Seven chromosome-wise significant SNPs (p < 0.05) were identified on chromosomes 4, 8, 14, 17, 18, 24, and 26 (Table 2). SNP OAR18_23478564.1 on chromosome 18 was significant for both additive and dominant models. Details on the function and location of the significant SNPs are in Table 2. One SNP on chromosome 4 (s55696.1) was located within a known ovine gene (*CPVL*). Two other SNPs were located within uncharacterised genes on chromosomes 14 (s53098.1) and 24 (s34109.1). The remaining SNPs were 25 to 456 kb away from the nearest gene. The proportion of the total variance explained by the significant SNPs was between 5 and 7%.

**Table 2:** **Chromosome-wise significant (p < 0.05) SNPs associated with footrot**

**Chr**	**SNP**	**Position (bp)**	Model*	**-log10 (p-value)**	**Variance explained** ^**‡**^	**Nearest gene**		**Function**
						**Name**	**Distance** ^**†**^ **(bp)**	
4	s55696.1	66980883	R	4.77	0.054	*CPVL*	between exons 2 & 3	carboxypeptidase, vitellogenic-like protein
8	OAR8_77678255.1	72284055	A	4.59	0.052	*HNRPDL*	455966	heterogeneous nuclear ribonucleoprotein D-like
						Uncharacterised (72529599–72531004)	−245544	unknown
14	s53098.1	8589373	R	4.50	0.051	Uncharacterised (8581480–8673246)	between exons 1 & 2	unknown
17	OAR17_29456634.1	26848221	R	4.52	0.051	Uncharacterised (26721069–26722410)	125811	unknown
18	OAR18_23478564.1	23886219	A	4.88	0.056	Uncharacterised (23697715–23776771)	109448	unknown
			D	5.65	0.065	*MORF4L1*	−288749	mortality factor 4 like 1
24	s34109.1	962868	D	4.35	0.049	Uncharacterised (961975–993337)	between exons 8 & 7	unknown
26	OAR26_8299529.1	6166783	R	4.50	0.051	*GPM6A*	24644	glycoprotein M6A
						Uncharacterised (6232840–6233142)	−66057	unknown

† Positive value denotes the gene located downstream of SNP, negative value denotes the gene located upstream of SNP.

‡ proportion of variance explained by the SNP.

* A – additive; R – recessive; D – dominant model.

## Discussion

Our aim was to perform an association analysis for footrot using dense SNP information. To our knowledge, this is the first attempt of using SNPs for association analysis of footrot in sheep. The most recent genome map for *Ovis aries* [33] was used to improve identification of candidate genes for the analysed trait. Although annotation of the sheep genome is good, there are still some regions that remain unknown which makes it difficult to identify candidate genes in all the regions that contain significant SNPs. It should be mentioned that, based on the current study, associations with candidate genes can only be treated as an indication and certainly need further research to be validated. Moreover, it is likely that the detected effects and variance explained by the significant SNP are overestimated. Given the small sample size, power to detect associations was low, which in consequence might have led to a situation where the detected effects were overestimated because such overestimated effects have a higher probability of being detected [38]. Moreover, the effect of the significant SNPs could be inflated due to the fact that animals with extreme trait values were sampled. However, these extreme animals were combined with animals with intermediate trait values, which should limit the overestimation of SNP effects. Linkage analysis could be considered as one of the solutions to increase the power of the analysis. However, for successful detection of (quantitative trait loci) QTL, an appropriate population structure with large full-sib or half-sib families is required [39,40]. Therefore, the small size of the families in the current study prevented us from applying this method. De-regressed EBV were used as pseudo-phenotypes in order to maximise the use of available information on animals that had been scored two or three times and had offspring with footrot scores. It should be noted that reliability of the EBV was limited to a certain extent due to low heritability of footrot and number of animals scored. Use of raw phenotypes could be considered as an alternative to the de-regressed EBV [41].

In the current study, we found no genome-wise significant SNPs but seven SNPs were significant on a chromosome-wise level. One of these SNPs was located in a known ovine gene, and three were located within uncharacterised genes. Unfortunately, the function of those genes has not been extensively studied and, currently, it is impossible to suggest a link between their functions and their potential role in the development of susceptibility/resistance to footrot. Therefore, based on the current analysis, it can be concluded that footrot resistance is not driven by single genes with a major effect. Most of the studies related to the sheep immune system are based on low-resolution genome screens, often resulting in very wide confidence intervals. This is the first time that a GWAS was performed for footrot in livestock. Therefore, considering the relatively small sample size, these results should be treated as a first step in unravelling the genetic background of footrot. Since no major genes responsible for footrot were identified, it can be hypothesised that this trait has a polygenic determinism. It is also worth noting that we found no significant SNPs in the MHC region, which could be due to the small sample size which inevitably reduced the power to detect significant associations. Similar problems have been reported in the case of QTL scans for nematode infections in sheep [42]. The extreme complexity of the MHC region has been reported to pose challenges in GWAS studies because associations may be within an extended haplotype that spans hundreds of genes [43]. An additional layer of complexity comes from alternative splicing which can be haplotype-specific [43]. These factors make it difficult to detect significant associations within the MHC complex using standard SNP association methods [41].

Additional analyses are required to increase our knowledge and understanding of the genetic determinism of footrot susceptibility. Future analyses will involve further GWA studies to refine the current results, as well as implementation of genomic selection for footrot. For this purpose, 3500 additional animals with footrot scores will be genotyped. These will serve as the reference population to estimate genomic breeding values. Given the low heritability and polygenic nature of footrot, it is expected that genomic selection will increase selection response in comparison to traditional best linear unbiased prediction (BLUP)-based selection [44]. Potentially, the use of the new high-density chip with 600 K SNPs will increase the power to detect genes that affect susceptibility to footrot infection [45] especially when combined with partial sequence analysis. The ability to characterise significant loci for footrot resistance would reduce the need to score large numbers of animals to ‘retrain’ the prediction equations for genomic breeding values. This would be beneficial particularly in dry years when footrot prevalence can be very low, yet more resistant animals could still be identified.

Linkage disequilibrium in the investigated population of Texel sheep was analysed because it has a strong influence on the power to detect QTL and accuracy of genomic selection [46,47]. The extent of LD found in the current population of Texel sheep is higher than that reported in Spanish Churra sheep (r^2^ of 0.15 for an average distance between SNPs of 40 to 60 kb) [48] and French Lacaune dairy sheep (r^2^ of 0.13 for an average distance between SNPs of less than 50 kb) [49]. Mean LD at 50 kb (average distance between SNPs on the 50 K chip) is slightly higher than that found in the French (r^2^ of 0.14 to 0.17) [50] and UK (r^2^ of 0.18) dairy goats [51]. The average LD estimated for the population of Texel sheep analysed here appears to be within the range of values reported for dairy cattle (r^2^ of 0.20 to 0.23 for an average distance between SNPs of 40 kb) [52-54] but is lower than those in pigs (r^2^ of 0.47 to 0.49 for an average distance between SNPs of 30 kb) [55]. The fact that the extent of LD in the current Texel sheep population is higher than that found in other sheep populations should increase the power to find significant associations. Further improvement could be achieved by using the new 600 K HD chip which has a much more dense distribution of SNPs across the genome and thus, fills the gaps in some of the regions where SNP spacing is large (>50 kb) on the 50 K ovine chip. The shape of the LD decay curve was analysed to make inferences about population history with respect to the recent and historical effective population size. The LD for small SNP distances was smaller than that expected for a population with Ne of 250, which indicates that, historically, the Texel population harboured higher levels of diversity. This is similar to the results reported for the French Lacaune sheep, for which the historical Ne also indicates that the original population was very heterogeneous [49].

## Conclusions

This study found seven SNPs significant on a chromosome-wise level. No major genome-wise significant QTL were identified. The current analysis did not identify any potential candidate genes for footrot susceptibility. Our results suggest there are no major genes responsible for this trait, which probably has a polygenic determinism. This study should be treated as an initial attempt to go some way towards identifying genomic regions that are associated with the development of footrot susceptibility/resistance in sheep.
